# HIV-Associated Tuberculosis in the Newborn and Young Infant

**DOI:** 10.1155/2011/354208

**Published:** 2011-04-04

**Authors:** M. Adhikari, P. Jeena, R. Bobat, M. Archary, K. Naidoo, A. Coutsoudis, R. Singh, N. Nair

**Affiliations:** ^1^Neonatal Team, Nelson R Mandela School of Medicine, University of KwaZulu-Natal, P.O. Box 17049, Congella, Durban 4013, South Africa; ^2^Department of Paediatrics, Nelson R Mandela School of Medicine, University of KwaZulu-Natal, P.O. Box 17049, Congella, Durban 4013, South Africa; ^3^Infectious Diseases Team, Nelson R Mandela School of Medicine, University of KwaZulu-Natal, P.O. Box 17049, Congella, Durban 4013, South Africa

## Abstract

Each year, approximately 250 000 women die during pregnancy, delivery, or postpartum. Maternal mortality rates due to tuberculosis (TB) and HIV in Sub-Saharan Africa now supersede obstetric-related causes of mortality. The majority of cases occur in population-dense regions of Africa and Asia where TB is endemic. The vertical transmission rate of tuberculosis is 15%, the overall vertical transmission rate of HIV in resource-limited settings with mono- or dual-ARV therapy varies from 1.9% to 10.7%. If the millennium development goals are to be achieved, both HIV and TB must be prevented. The essential aspect of TB prevention and detection in the newborn is the maternal history and a positive HIV status in the mother. Perinatal outcomes are guarded even with treatment of both diseases. Exclusive breast feeding is recommended. The community and social impact are crippling. The social issues aggravate the prognosis of these two diseases.

## 1. Burden of Disease

Each year, approximately 250 000 women die during pregnancy, delivery, or postpartum. Maternal mortality rates due to tuberculosis (TB) and HIV in Sub-Saharan Africa now supersede obstetric-related causes of mortality [1].

There has been a global increase of tuberculosis in women due to the intersecting epidemics of HIV and TB. Since 1990, the number of TB cases has quadrupled in some regions of Africa and 75% of TB patients are HIV infected [2].

HIV and TB affect women in the prime child bearing age range of 15–29 years, contributing to an increased mortality in this age group [3].

TB and HIV are independent risk factors for maternal mortality. In Sub-Saharan Africa, 3-4% of HIV infected mothers die within a year of parturition [3–5]. 

 A significant increase in tuberculosis in pregnant women was shown by an increasing caseload from 0.1% to 0.6% between 1996 and 1998 in Durban South Africa. Of these mothers with TB, 115 (78.8%) were HIV infected, 26 (17.8%) were uninfected. The minimum TB/HIV coinfection rates increased from 36.4% in 1996 to 88.3% in 1998 (*P* < .001). The TB prevalence rate for HIV noninfected maternities at King Edward VIIIth Hospital Durban was 72.9/100 000 and for the HIV infected maternities 774/100 000 with a relative risk of TB due to HIV infection of 10.62 [6]. The mortality rate in the TB infected mothers was 103/100 00 with 14 of the 15 mothers who died being HIV infected [6]. 

Epidemiological indicators of TB indicate that the Millennium Development Goal of TB elimination by 2050 is not likely to be achieved. The majority of cases occur, as indicated above, in population-dense regions of Africa and Asia where TB is endemic. The persistence of TB in the setting of poor existing health infrastructure has led to an increase in drug-resistant cases, the majority associated with HIV coinfection [7].

Unfortunately HIV influences the presentation of TB in a number of ways. Smear negative cases are more common in those who are HIV infected [8]. In addition, the lower bacterial load and the atypical chest radiographic findings add to the difficulty of arriving at a diagnosis of TB [9]. The pathological features of TB in HIV-infected women differ from those without HIV. The TB granulomas in the lung of HIV-positive individuals show extensive necrosis, poorly formed granulomas, and an infiltration of polymorphonuclear cells. TNF was greatly reduced in TB/HIV co-infected individuals suggesting that HIV through TNF alters the immune response leading to impairment of the antituberculosis response [10]. In TB meningitis HIV positive individuals have reduced inflammatory responses and extensive vasculitis [11].

## 2. Vertical Transmission of TB

The fetus and newborn of the pregnant woman with TB are exposed to the disease and may become infected. The extrapulmonary forms of the disease, miliary and meningeal TB, are greater risk factors for congenital TB [8]. On the other hand, vertical transmission did not occur if mothers had pleural effusion, generalized adenopathy. 

In Durban, South Africa 15% of the mothers with TB transmitted the organism to their infants in the first three weeks of life. Mothers with TB diagnosed prenatally, transmitted TB in the first week of life. Infants whose mothers with TB diagnosed in late pregnancy or the immediate postpartum period presented with TB in the third week of life. Mothers who did not receive prenatal care transmitted TB more readily to their infants as did mothers who were nonadherent to TB therapy [12].

## 3. Vertical Transmission of HIV

The vertical transmission rate of HIV documented in a study conducted in Durban South Africa in the 199Os was 34% [13].

In current studies of routine neonatal followup of 588 well term infants at a local clinic, the PCR at six weeks indicated the overall transmission rate was 10.7% (63/588), with dual therapy (maternal AZT antenatally and a single-dose nevirapine at the onset of labour, and baby receiving a single dose of nevirapine, the transmission rate and PCR testing at 6 weeks and 9 months was 1.9% (3/154). In mothers and babies taking a single dose of nevirapine only the transmission rate was 12.8% (49/382), and if no ARV, AZT, or nevirapine was taken, the transmission rate was 22.4% (11/49). Dual therapy resulted in an 82% reduction in transmission (RR 0.18 [CI 0.07–0.49] Maternal ARV exposure and transmission rate was highly significant (*P* < .0001) [14]. 

Preliminary results in a follow-up study of 457 high risk infants both term (birth weight >2500 gms, *n* = 165) and low birth weight (birth weight <2500 gms, *n* = 292) from King Edward VIIIth Hospital, Durban South Africa, 2 (0.4%) of the term infants died and 11 (2.4%) of the low birth weight infants died. The HIV transmission rate in the term infants was 9.1% and in the low birth weight were 13%. Some of the mothers were on monotherapy and some on dual therapy. The mean CD4 count of the mothers whose babies died and in those babies that required ARVS based on positive PCRs at 6 weeks was 303 cells/mm^3^ [15].

Effective prevention of mother-to-child transmission (PMTCT) strategies can now decrease HIV transmission to less than 1%, not only during the intrauterine and intrapartum period but also during breastfeeding [16]. The most efficacious method of ensuring both optimal maternal health and reducing transmission risk to the infant is the use of maternal HAART [17]. However, the use of HAART in pregnancy is associated with an increased incidence of preterm delivery and low birth weight, which requires monitoring during the pregnancy [18].

The effect of HIV-associated immunodeficiency is marked in infants as demonstrated by blunted Cytotoxic T-lymphocyte (CTL) responses in HIV-infected infants particularly if infected early during the pregnancy [19]. These immunological effects are manifested by decreased CD4 counts in both premature and term infants at birth and by a rapid decline in the CD4 count over the first 6 months of life [20, 21].

The above unpublished studies in low birth weight infants need to be verified in further studies.

## 4. Clinical Features of TB and HIV in Pregnant Women

TB in pregnant women presents with the same clinical features as the nonpregnant women [22, 23]. 

The clinical spectrum of the signs of TB may vary from mild cough, fever, and fatigue to overt signs of weight loss and gross radiological changes [24]. The diagnoses of TB and HIV may be delayed by the common symptoms of malaise and fatigue of pregnancy. Extrapulmonary TB occurs in 10–15% of pregnant women with TB [25]. The chest radiograph may reveal the radiological features of TB in an asymptomatic mother [26]. The crucial aspects of the diagnosis are a previous history of TB, current history of TB receiving therapy, a contact at home, persistent cough for two weeks or more, and an unresolving pneumonia. 

Maternal genital TB is subclinical and may be considered when there is no overt TB in the mother and TB is diagnosed in the baby [6]. 

Tuberculosis and HIV in pregnancy may have three presentations; firstly, a pregnant woman develops tuberculosis and is found to be HIV infected and therefore will require treatment for both diseases; secondly, tuberculosis is diagnosed in an HIV-infected mother who does not herself require antiretroviral therapy but for the prevention of mother-to-child transmission of HIV; thirdly overt signs of TB may appear in an HIV-infected pregnant woman who has commenced HAART [24]. This may be an immune reconstitution infection syndrome (IRIS) following exacerbation or aggravation of underlying TB [27].

## 5. Clinical Features of Tuberculosis in the Newborn Period

The essential aspect of TB prevention and detection in the newborn is the maternal history. A positive HIV status in the mother increases the likelihood of TB in the mother [28, 29]. The critical points in the maternal history are given in the section above “Clinical Features of TB and HIV in pregnant women”. Transmission and outcomes are definitely affected by duration of therapy before delivery. Four months or more of therapy is protective to the fetus. However, noncompliance with therapy carries an increased risk of transmission of the *Mycobacterium tuberculosis* to the infant [6]. 

Symptoms of tuberculosis in the neonate are usually in the low birth weight, growth-retarded infant and are nonspecific including lethargy, poor feeding, poor weight gain and unresolving or recurrent pneumonia. Signs and symptoms may be present at birth but overt expression of the disease more often occurs in the second or third week of life when respiratory problems, hepatosplenomegaly, and lymphadenopathy are present [30]. Other clinical features may include skin lesions, seizures, jaundice, ear discharge, paravertebral abscess, and haematological anomalies and ascites. Although the signs may suggest chronic intra-uterine infection and they may also mimic acute infection, depending on the clinical phase and severity of the disease. The diagnosis may be difficult and therefore missed [31]. In a setting of poor response to antibiotics and supportive therapy and when microbiological evaluations for acute infections and serological tests for chronic intrauterine infections are negative, tuberculosis should be considered, particularly if the mother is known to be infected with HIV. 

The above physical signs of chronic intrauterine infection may represent the features of TB and coinfections such as cytomegalovirus infection setting a stage for rapidly progressive disease [32].

## 6. BCG and the HIV-Infected Infant

BCG is a live attenuated vaccine derived from Mycobacterium bovis. The vaccine is given to infants at or around birth to provide protection from severe TB disease during childhood (including TB meningitis and miliary TB) however it gives no protection against lung disease. Several distinct strains of BCG derived from the original BCG strain are used in immunization programs around the world, each with varying immunogenicity and side effect profile. With over 90% estimated vaccine coverage, especially in TB endemic regions, BCG is the most frequent administered vaccine globally with over 100 million doses administered annually [33]. 

With the overlap of high TB and HIV prevalent countries, BCG is also given in areas of high HIV incidence. While the risks of adverse events associated with BCG vaccination in HIV-negative infants are low (<0.04% for local disease and 0.0002% for disseminated disease), HIV-positive infants have a markedly increased risk of developing local (5.6%) and disseminated BCG disease (BCGosis) (0.2%) [34]. The World Health Organization (WHO) has therefore recommended that BCG be only given to HIV-negative infants, however implementation of this recommendation is impractical when BCG is given at birth and the HIV status has not been determined.

Clinical features associated with BCG disease include local ulceration at the injection site, ipsilateral axillary lymphadenitis, and abscess formation and disseminated disease with involvement of the lung, liver, and bone marrow. Disseminated disease in HIV positive infants has a 75% mortality [35].

Management of BCGosis is a combination of antituberculosis drug therapy. BCG is resistant to pyrazinamide and some strains are resistant to isoniazid (INH). 

Drug therapy should include ARVSs and high dose INH, Rifampicin, ethambutol, and a fluoroquinolone for a minimum of 9 months. Careful monitoring for toxicity and clinical response to treatment are important.

## 7. Investigation of the Neonate

Criteria for investigation of TB are based, as indicated above, on the maternal history, the physical signs in the newborn, the failure to respond to antibiotics, and supportive therapy and maternal HIV positivity [6].

Investigations are from multiple sites and may have to be repeated. The chest radiograph is most often atypical of TB and may be difficult to interpret. Repeated radiographs may show a more classical picture.

Specimens from the neonate suitable for microscopy and culture include gastric aspirates, induced sputum, tracheal aspirates if the neonate is mechanically ventilated, cerebrospinal fluid and, if seen on the chest radiograph, pleural fluid. Early morning gastric aspirates should be taken on three consecutive days before the first feed of the day [24]. If deemed appropriate, a liver or lymph node biopsy may be undertaken for histology and culture of *M. tuberculosis*. In the event of death, relevant biopsies (e.g., liver, lung, nodes, and skin lesions) should be taken, with maternal consent, for histological and microbiological examination. Samples are examined microscopically for acid fast bacilli and cultured on standard media for 12 weeks [30]. If a neonate has skin lesions or an ear discharge, specimens should be taken for microbiological examination [31]. Induced sputum is more likely to be positive on microscopy and culture than gastric aspirates [36]. Broncho-alveolar lavage (BAL) has been recognised as an important investigation in children and this has been extrapolated to the newborn [37]. Detection of *M. tuberculosis* DNA in BAL fluid by polymerase chain reaction (PCR) has been shown to be of value [38]. Newer more rapid tests for the diagnosis of tuberculosis are urgently needed. The GeneXpert is a new test marketed to detect active TB and rifampicin resistance within 90 minutes. This test will be of importance in communities with a high burden of tuberculosis including MDR TB. However the test is expensive and has to be made available at a lower cost [39].

Line probe assay is a rapid method of detecting sensitivity in source cases for sensitivity [40].

## 8. Investigations for Tuberculosis of the Newborn

- Chest radiograph.
- Three early morning gastric aspirates/washings.
- Induced sputum.
- Endotracheal aspirate (if baby is mechanically ventilated).
- Cerebrospinal fluid.
- Blood culture for *Mycobacterium tuberculosis. *
- Liver biopsy when indicated.
- Lymph node biopsy when indicated.
- Cultures of skin lesions or ear discharges.
- Bronchoalveolar lavage (BAL).
- Polymerase chain reaction (PCR) on BAL fluid.
- Future tests GeneXpert
- Line probe assay.

In all babies who are exposed to or are diagnosed with TB, HIV must be excluded. The cheapest is to test the mother by the HIV Elisa. If the mother is HIV positive, infection should be confirmed in the baby with the DNA PCR which has a sensitivity of 38% at birth and 100% by two months and six months. RNA PCR sensitivity is 47% at birth and 100% sensitivity at 2 months and 6 months [41].

## 9. Investigation of the Mother

Pregnant women with TB undergo the same investigations as nonpregnant women and a HIV Elisa has to considered [20].

## 10. Management of the Baby with TB and HIV

### 10.1. Management of the Asymptomatic Newborn (TB)

If the newborn is asymptomatic and the mother is considered to have the sensitive mycobacterium, the infant should receive prophylaxis as soon as the investigations have been completed. Prophylaxis is isoniazid (INH) 10 mg/kg/day and rifampicin 10 mg/kg/day for 3 months [40]. The Bacille Calmette-Guérin vaccine (BCG) is withheld until completion of the period of prophylaxis. However, in some situations where followup is not certain the BCG is given on discharge from the neonatal unit (see above on BCG). At the end of the 3 months the chest radiograph should be repeated. In addition, the baby should be screened for other coinfections such as cytomegalovirus, herpes simplex, and herpes zoster. If, during the period of prophylaxis, the infant develops signs that suggest TB or if the cultures are positive, full anti-TB therapy should be commenced and continued for 6 months. In HIV and TB high prevalence areas, prophylaxis may not offer protection against acquisition of TB. If the maternal HIV status is unknown the mother should be tested.

### 10.2. Management of the Symptomatic Newborn

A differential diagnosis of HIV, syphilis, cytomegalovirus, congenital herpes, and atypical pneumonia (*Mycoplasma pneumoniae*) should be considered in infants who present with the physical signs indicated above [42]. On clinical suspicion, anti-TB therapy should be commenced once the relevant samples for tuberculosis have been taken. 

Standard WHO, recommended drug regimens are INH 10 mg/kg/day, RIF 10 mg/kg/day and pyrazinamide 20 mg/kg/day for two months followed by a four-month course of INH and RIF [42]. Therapeutic response is determined by the regression of clinical signs, weight gain, improved appetite, and radiological resolution where applicable.

If the baby is HIV PCR positive, following two weeks of anti-TB therapy or longer and depending on the clinical condition of the infant and there are no side effects of treatment, ARVs may be commenced. The process of commencing ARVs is very difficult, particularly if the mother is not on therapy. Practical issues of giving the infant many medications are experienced by the nursing staff and the mother. The mother has to be in a clinically reasonable condition to manage her baby. It is important to train another individual in the household to assist the mother. Experience with respect to the ARVs and their dosages in the newborn are increasing. 

Zidovudine (AZT: nucleoside reverse transcriptase [NRTI]) is the ARV that has been most studied in the newborn followed by lamivudine [43]. Zidovudine is given at a dosage of 2 mg/kg/dose four times per day, lamivudine (3TC NRTI) at 3 mg/kg/dose twice daily and lopinovir/ritonovir (Kaletra Protease Inhibitor [PI]) at 300 mg/m^2^ twice daily. Ritonovir boosts the lopinovir level. These dosages are according to the reference given and which is currently prescribed in South Africa. This may change in a short space of time.

## 11. Management of the Mother with TB and HIV

Once TB is diagnosed in any of the clinical settings, prompt anti-TB therapy has to be commenced in order to prevent the adverse effects in the mother, fetus, and newborn. The aim is to render the mother non-infectious [44–47].

Treatment of TB in pregnant women follows the WHO recommendations and is the same as for the nonpregnant based on standard therapy for 6 months and, in coinfection with HIV prolonged to a minimum of 9 months. The first line drugs, isoniazid, rifampicin, ethambutol, and pyrazinamide in standard doses, are readily absorbed from the maternal gastrointestinal tract and freely cross the placenta. The CDC recommendation for treatment of TB in pregnancy is isoniazid (INH) rifampicin (RIF) ethambutol daily for two months followed by INH and RIF daily or RIF and ethambutol daily for 4 months or twice weekly for 7 months, for a total of 9 months treatment in the latter group. Pyrazinamide (PZA) is not recommended as its effects on the fetus are unknown. HIV-infected women should receive RIF and although the routine use of PZA in pregnancy is not recommended (in the USA), the benefits of PZA in the HIV-infected woman outweigh the unknown risks to the fetus [48–50]. Streptomycin should not be used due to the harmful effects on the fetus. 

The recommendation for HIV positive pregnant women with pulmonary TB classified as WHO clinical stage 111 and 1V are eligible for ARV therapy irrespective of CD4 lymphocyte counts. WHO clinical stage 1 and 11 are offered ARV therapy if the CD4 count is <200 cells/m^3^ [27]. The trend now is towards earlier ARV therapy and most guidelines recommend treatment when the CD4 count is <350 cells/m^3^ [2]. All women with stage 3 or 4 irrespective of CD4 count [51].

In resource limited settings women with satisfactory CD4 counts are offered zidovudine from 28 weeks and nevirapine at the onset of labour as prophylaxis against mother-to-child transmission of HIV. In developed countries it is standard practice to offer ARVs aiming at undetectable viral load before delivery.

ARVs with concomitant TB therapy are complicated by a high pill burden, drug toxicities, drug interactions, and the immune reconstitution syndrome (IRIS). ARV therapy may exacerbate or aggravate underlying TB (reported in 20% patients commencing ARV therapy). IRIS is more likely to occur during the first two months of commencing ARV therapy if CD4 counts are <100 cells/m^3^ [27]. 

In resource-restricted settings, the first line regimen of NRTIs and one nonnucleoside reverse transcriptase inhibitors (NNRTI). The preferred drugs, cheaply manufactured as a fixed dose tablet taken twice a day, are stavudine (40 mgs twice daily), lamivudine (300 mgs daily), and nevirapine (200 mgs daily for two weeks then 200 mg twice daily). Stavudine is less favoured and options include substitution of zidovudine for stavudine and efavirenz for nevirapine.

WHO guidelines for 2010 include the following possible combinations:
zidovudine + lamivudine + nevirapine,
tenofuvir + lamivudine (or emotricitabine)+ NVP
zidovudine u lamivudine + efevirenz, or
tenofuvir + lamivudine (or emtricitabine) + efavirenz.

Efavirenz is not recommended for the first trimester of pregnancy. The newborn of the mother on ART whether breastfeeding or giving replacement feeds receives zidovudine twice daily or nevirapine daily for four to six weeks of age [51]. 

Drug interactions between ARVs and TB therapy pose problems. RIF induces CYP450 which leads to a reduction of the plasma concentration of nevirapine by 30% and efavirenz by 20–25%. The clinical significance of this is not known but there are concerns that such reduced nevirapine levels may induce drug resistance and treatment failure. However, increasing nevirapine dosage for compensation for plasma reduction, increases the risk for toxic effects of the drug, particularly hepatotoxicity [52]. The combination of RIF and nevirapine should be avoided particularly in pregnancy. However, it has been shown that in patients with a low CD4 count the risk of hepatotoxicity is reduced. WHO recommends abacavir or tenofovr instead of nevirapine but this is an expensive option. The advantages outweigh the disadvantages. It is suggested that RIF should be used in the initial phase of TB therapy, isoniazid and ethambutol is continued and HAART with nevirapine may then be commenced. Efavirenz, much more expensive, should be avoided in pregnancy due to concern about teratogenicity. The duration of TB therapy in a HIV positive patient may have to be extended to 9 months duration and there is an ongoing risk of “second episode” of TB [40]. It is now considered prudent to treat both TB and HIV at the same time to avoid unnecessary deaths due to delay in commencing therapy [53].

## 12. Multidrug Resistant Tuberculosis

Drug resistant TB is increasing worldwide as was noted by Edlin et al. [54]. MDR TB as a cause of death in patients co-infected with TB and HIV is well recognized [55].

Multidrug resistant (MDR) TB defined as resistance to INH and RIF was identified among hospitalised patients with AIDS. Ninety to 98% of patients with MDR TB are HIV co-infected [56]. Resistance may be primary and occurs when an individual is infected with *Mycobacterium tuberculosis* which is already resistant to a particular drug; secondary resistance is when drug-resistance organisms emerge as the dominant population during treatment. Risk factors for the development of MDR TB are poor adherence and compliance to therapy, inadequate assessment of response, incorrect dosage or number of drugs used as prophylaxis or treatment, previous prolonged treatment of TB, contact with a patient known to have drug resistant disease HIV infection and drug abuse. Patients suspected of MDR TB should be referred to a tuberculosis centre specialized in this condition.

The side effects of drugs lead to a risk of noncompliance and nonadherence in therapies for both diseases increasing the risk for the pregnant woman and her fetus/child. The burden of pills and duration of therapies in itself poses logistical issues, MDR TB having to be treated for two years. Suspected MDR TB in a pregnant woman should be treated with INH, RIF, Ethambutol, and a quinolone until culture and sensitivities are available. The newborn baby should be given prophylactic therapy of high dose INH (15 mg/kg/day), ethionamide (10 mg/kg/day), and a quinolone (10 g/kg/day) [57]. The aminoglycosides, capreomycin, pyrazinamide, and quinolones are contraindicated in pregnancy. Newer therapies include rifamycins, macrolides, clofazimine, amoxicillin and clavulanic acid [58]. 

Reports of the safety of second-line drugs for MDR TB in pregnancy have appeared, but very small numbers of children have been followed, 7 in the short term and 6 children for long-term evaluations [59]. The drugs appear to have no adverse effects on the children and none had acquired TB [58, 60].

In our setting a few pregnant women have had MDR TB, so far their babies have been TB negative. WHO is in the process of developing further guidelines for TB, MDR TB and congenital TB which may change current approaches to therapy. From India guidelines have been summarized for the management of pulmonary TB, extrapulmonary TB, and TB in special circumstances [61].

## 13. Outcomes

### 13.1. Maternal Mortality

Maternal mortality in relationship to HIV and TB was discussed in the introduction. The following studies examine the causes of maternal death.

In a two-year retrospective study of maternal deaths at a teaching hospital in Lusaka, Zambia, 58% of the deaths were due infections, malaria (30%), TB (25%) respiratory infections (22%), and other HIV-associated opportunistic infections [62]. In a hospital-based, five-year study at tertiary level in Johannesburg, South Africa, 106 maternal deaths occurred in 36708 deliveries. Seventy percent of deaths were HIV related, mainly TB and pneumonia. Maternal mortality ratios in HIV infected women were 776 per 100 000, 6.2-fold higher than HIV negative women, 124 per 100 000 [63].

In an autopsy study of 171 maternal deaths in Mozambique, 56% of the deaths were due to nonobstetric causes, mostly HIV/AIDS, related infections, lung sepsis, meningitis, malaria, and TB [64].

### 13.2. Obstetric, Perinatal, and Neonatal Outcomes

In earlier studies on TB in pregnancy there was no significant effect on outcomes [64]. This has changed due to the impact of the HIV epidemic [2, 3]. The effects may be more significant in low socioeconomic populations. If diagnosed and treated, the effects of tuberculosis on pregnancy and the neonate are not so serious but in populations with a low socioeconomic status tuberculosis increases the risk of abortions and premature delivery [65]. In studies from India, the outcome of pulmonary tuberculosis in 79 pregnant women compared to 316 cases matched for age and socioeconomic status revealed infants born to women with tuberculosis were significantly lighter than the controls; there was a two-fold increase in prematurity (22.8% versus 11.1%), a small size for gestational age (20.2% versus 7.9%), and a 6-fold increase in perinatal deaths (10.1% versus 1.6%). The adverse effects were more pronounced in those cases in which tuberculosis was diagnosed late in pregnancy, when adherence to antituberculosis treatment was poor and in those with advanced pulmonary disease [44]. A similar study on 33 pregnant women with extrapulmonary tuberculosis, showed that disease confined to lymph nodes was not associated with adverse obstetric outcomes but that those with more serious manifestations of disease (intestinal, skeletal, meningeal, renal, and endometrial) suffered similar adverse outcomes as those with pulmonary tuberculosis [7].

In Mexico, late diagnosis and poor care resulted in a 4-fold increase in obstetric morbidity and a 9-fold increase in preterm labour [46]. In a further study from that country, the perinatal outcomes of 35 mothers with tuberculosis were compared to those of 105 healthy mothers and revealed that, in the former, the neonates were significantly smaller (2.8 versus 3.1 kg) and that the relative risks of premature delivery and perinatal death were, respectively, 2.1 and 3.1. Poor outcomes were particularly prominent in women with advanced pulmonary tuberculosis in late pregnancy [44]. In Durban, South Africa, neonates of women with tuberculosis were significantly smaller, with 49% having a birth weight less than 2.5 kg compared to 21.8% for the overall hospital deliveries [6].

Perinatal mortality in those mothers with tuberculosis and HIV was 1.6-fold higher than for the King Edward hospital, Durban and the KwaZulu Natal region. 

Pregnancy in women infected with HIV-1 alone tends to result in low birth weight premature babies, particularly in those with advanced HIV disease [48]. The outcome of pregnancy in those with both HIV disease and tuberculosis is worse, but it is not entirely clear which disease has the greater bearing on adverse outcomes. In the South African study, followup of those infants with tuberculosis and HIV infection showed persistent or worsening clinical signs, with death occurring on average by the age of 9 months [32]. At this time ARVS for the prevention of mother-to-child transmission had not been implemented. Nevirapine prophylaxis was introduced in 2001.

## 14. Rapidly Progressive Disease

Coinfections set the stage for rapid progression of HIV disease. This was shown in babies who were HIV exposed and had tuberculosis. Treating the TB did not reduce the mortality from HIV and all babies died by nine months of age [32].

Another important factor is the immunological status of the mother. Intrapartum transmission occurred in mothers with very high viral load compared to mothers with intrauterine transmission (5 160 000 versus 984 000 copies/ml). Single-dose nevirapine impacts on intrapartum transmission. However disease progression was rapid with 85% of the infants requiring ARV therapy within six months [20]. 

It is important to recognize the early clinical manifestations of HIV-infected infants in the first two months of life in order to prevent the disease progressing rapidly, to undertake the HIV DNA PCR, and to commence ARVs early [66]. The uninfected infant remains at risk of more frequent infections and suffers morbidity greater than the unexposed infants in early infancy [67].

## 15. Breastfeeding or Formula Feeding

Breast feeding is recommended by the American Academy and the Revised National Tuberculosis Control in India [67, 68]. Tuberculosis is transmitted by the aerogenous route; the mother with open tuberculosis can still infect her baby if she formula-feeds her baby. However, the risk of transmission through breast milk is negligible and the baby should be on either prophylaxis or treatment for TB. In resource-poor settings exclusive breast feeding has to be emphasized. However, in the more advantaged setting, mothers can choose replacement feeding. The first-line anti-TB agents are excreted in milk in very small amounts have no adverse effects, and do not seem to predispose to the development of drug resistance.

Breast milk carries a risk of transmission of HIV, however, exclusive breast feeding reduces HIV transmission when compared to mixed feeding [68]. In addition, mixed feeding carries a risk of increasing diarrhoeal and respiratory infections [69]. The above is of relevance in underprivileged communities. The risks of a higher infant mortality, severe pneumonia and diarrhea by 6 months of age on formula feeding were shown in the MASHI study [70]. However, in settings where replacement feeding is feasible, affordable, and safe, HIV-infected women should be encouraged to exclusively formula feed. This follows the WHO recommendations [69].

Further support for women who are HIV positive and who wish to breastfeed, comes from the studies indicating that flash heating of maternal milk inactivates HIV with about a 20% loss of proteins and antibodies [70]. This method has been shown in the neonatal unit at King Edward VIIIth Hospital, Durban to be feasible and practical, some mothers are undertaking flash heating at home. The method is a simple one of expressing breast milk into a “jam jar”, closing it, placing the jar in a pot of cold water with the level of the water two finger breadths above the level of the milk in the jar, remove the lid of the jar, heat the water to a rapid boil, and remove the jar. The milk is allowed to cool for 10–15 minutes and may then be given to the baby. Milk not used immediately may be be placed in the refrigerator and used at the next feed. The jar has to be carefully closed and labeled with name, date, and time.

A number of studies have shown the reduction in HIV transmission during breast feeding using different combinations of ARV therapies. 

Extended ARV prophylaxis with NVP dual therapy with NVP and AZT for 14 weeks of life significantly reduced breast milk transmissions [71]. Kilewo et al. showed that maternal HAART for 6 months and infant NVP for 6 months were equally efficacious in reducing postnatal transmission through breast milk at 6 months. International recommendations on breastfeeding in HIV positive women may not be recognized or accepted by health workers undertaking the relevant counseling [72].

With the access to treatment with ARVs during pregnancy and the postpartum period of the newborn, the risks of HIV transmission through maternal breast milk are negligible [73]. It is with concern that in the Neonatal Unit at King Edward VIIIth Hospital Durban, South Africa, over a 6-month period in 2009, from routine morbidity and mortality records, it was noted that 10 very low-birth-weight infants suffered necrotizing enterocolitis. All but one were formula-fed, all were HIV exposed, two babies had gut perforations, and one died. Three of the mothers were on ARVS believing that the ARVs would protect their babies.

## 16. Social Issues

Social issues impact heavily on the management of HIV and TB. The lack of adequate caregivers and maternal orphans are major problem. The risks of therapies which carry a high medication burden includes nonadherence, non compliance, and poorer outcomes for the infected infant [27]. 

Both HIV and TB are associated with stigma which has resulted in added suffering due to lack of disclosure, discrimination, and criminalization to mention the commonest associations with stigma. Stigma has seriously impeded the care of patients, increased morbidities, the implementation of programs and, in some circumstances, resulted in poor adherence [74, 75].

 In 2006, in South Africa, there was an estimated 122 000 children living in an estimated 60 000 child headed households; the majority of whom were in three provinces: Limpopo, KwaZulu Natal, and the Eastern Cape. Many live with grandparents with their own vulnerabilities. In some areas 45% of these children live with grand or great grandparents. These children are exposed to risks of poor education, little food, physical and sexual abuse, prostitution, and child labour [76].

## 17. Conclusion

The TB and HIV epidemics are a devastating combination of infections resulting in severe morbidities and mortality rates from these diseases as well as complications of the combination of therapies. The social issues paralyse family dynamics; stigma creates further dysfunctionality in society. The only hope is for PMTCT to be seen as the most critical aspect of HIV prevention; awareness of and early recognition of TB is the second hope which must be achieved urgently if the millennium development goals of reducing maternal, child, and TB mortality are to be achieved.
